# Analysis and mathematical modeling of the survival kinetics of *Staphylococcus aureus* in raw pork under dynamic and static temperature conditions

**DOI:** 10.1002/fsn3.2604

**Published:** 2021-10-08

**Authors:** Xue Bai, Ying Xu, Yong Shen, Na Guo

**Affiliations:** ^1^ College of Food Science and Engineering Jilin University Changchun China

**Keywords:** dynamic temperature, one‐step analysis, predictive model, raw pork, *Staphylococcus aureus*

## Abstract

The incidence of frequent foodborne disease outbreaks due to *Staphylococcus aureus* contamination necessitates the urgent searching for effective methods to monitor *S. aureus*. This research aims to construct model with a dynamic survival curve and some static growth curves to predict the behavior of *S. aureus* in raw pork. Lack of research about *S. aureus* kinetics in pork under fluctuating temperature conditions across freezing and thawing necessitates this study. One‐step analysis was used to determine the model parameters, which was more efficient than conventional model analysis with two steps. The results of kinetic analysis showed that *T*min (minimum growth temperature) was 6.85°C, which is close to the estimated values in previous reports. Subsequently, validation results indicated the integrated model can accurately predict the behavior of *S. aureus* regardless of isothermal or nonisothermal conditions with the root‐mean‐square errors (RMSE < 0.44 log CFU/g, 73.9% of the errors of prediction falls within ±0.5 log CFU/g), accuracy factors *A_f_* and bias factors *B_f_* were both close to 1. This work may offer an effective method for the assessment of microbial security related to *S. aureus* in pork.

## Graphical abstract

This research conducted predictive model with dynamic and static growth curves to predict the survival of *S. aureus* in raw pork under different temperature conditions. One‐step analysis was used for the model parameters determine, which is more efficient than conventional model analysis in two steps. The validation results indicated the integrated model can accurately predict the behavior of *S. aureus* regardless of isothermal or non‐isothermal conditions.

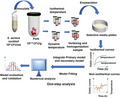

## 1 INTRODUCTION

The growing morbidity of foodborne illnesses induced by contaminated food, in particular meat and meat products, has received increasing attention according to reports from the European Food Safety Authority, the European Centre for Disease Prevention and Control, and the United States Department of Agriculture (Centers for Disease Control and Prevention (CDC), 2017; Food & Authority, 2018; Food & Authority, 2019; United States Department of Agriculture (USDA), 2017). *Staphylococcus aureus* are typical foodborne pathogens, which induce foodborne intoxication globally by the production of heat‐stable enterotoxin (Carvalho et al., 2021; Hani Tabaie Zavareh & Ardestani, 2020; Mama et al., 2020; Oliveira et al., 2020). As mentioned by some reports, a large number of staphylococcal food poisoning cases were brought about by contaminated meat products (Grispoldi et al., 2021; Lee et al., 2015). Thus, it is necessary to monitor the survival behavior of *S*. *aureus* under different conditions in pork products.

Numerous studies investigated the *S*. *aureus* growth behavior in meat or meat products under different temperature conditions. Mansur et al described the kinetic behavior for *S. aureus* in raw meat, ham, and sausage under isothermal (static) conditions (10, 15, 20, 25, 30, 35, and 40℃) (Mansur et al., 2016). Similarly, Lee et al. (2015) used a large number of growth curves under isothermal conditions to model the growth kinetics of *S. aureus* in pork. However, most of the researches were under the static temperature conditions (Kim et al., 2018; Lee et al., 2015; Mansur et al., 2016). Temperature fluctuations will occur during household storage and in the food supply chain when products are transferred from retail stores to lorries and customers (Oliveira et al., 2009). Freezing–thawing might also be involved in food supply (Teuteberg et al., 2021; United States Department of Agriculture (USDA), 2020). Thus, the impacts of fluctuating temperatures on the behaviors of pathogens should be recorded. The growth of *Escherichia coli O157:H7* and *Salmonella* in ground meat at dynamic temperature conditions have been analyzed (Hwang & Huang, 2018; Reddy et al., 2011). Nevertheless, none of the studies are concerned about how dynamic temperature variations across freezing and thawing affect *S. aureus* survival in raw pork. The lack of researches emphasizes the necessity of this work.

As a common mathematical analysis, predictive microbiology can be applied to predict the survival behaviors of microorganisms and mathematically model reproducible behavior under some environmental conditions, contributing to obtain information about the survival behavior of microorganism. The bacterial growth curves under isothermal conditions can be described by some main empirical models like the Gompertz model, logistic model, Baranyi model, and Huang model (Fujikawa et al., 2004; Li et al., 2013; Lu et al., 2020). The inactivation kinetics can be modeled using the Geeraerd and Mafart model (González‐Tejedor et al., 2018). During traditional modeling, a large number of isothermal curves and a two‐step approach for data analysis were used (Lee et al., 2015; Li et al., 2013). In the first step, a primary model as mentioned above was fitted with isothermal data and the kinetic parameters were estimated. In the second step, a secondary model was used for further analysis to find the impact of environmental factors (Kataoka et al., 2017; Rubio et al., 2018). However, this two‐step method is time‐consuming for large amounts of isothermal data collections and inefficient with higher errors. In this case, the one‐step analysis which integrates the primary model and secondary model into one step might provide a reliable approach (Huang, 2015; Huang & Hwang, 2017; Hwang & Huang, 2018; Li et al., 2019; Liu et al., 2020). The one‐step analysis can be used to construct a predictive model not only under isothermal (static) conditions but also under nonisothermal (dynamic) conditions via a numerical method. Liu et al. (2019) used the one‐step analysis to accurately predict *L. monocytogenes* growth in braised beef under fluctuating temperatures and static conditions, the reliability of the one‐step analysis method was hence validated. In addition, some researches indicated that the one‐step dynamic analysis could provide more accurate estimates with fewer tests than the static analysis for the microbial kinetic analysis (Cattani et al., 2016; Liu et al., 2019).

The objective of the research was to (i) obtain and determine the kinetics parameters with the one‐step approach under isothermal (static) combined with nonisothermal (dynamic) temperature conditions; (ii) construct a model to describe the survival kinetics of *S. aureus* in raw pork under various temperature conditions simulating temperature fluctuations in food supply chain and household storage; (iii) perform a validation and evaluation for the model with other curves which were set aside.

## 2 MATERIAL AND METHODS

### 2.1 Bacterial strains and culture conditions

*Staphylococcus aureus* ATCC29213 (China Medical Culture Collection Center, Beijing, China) and three foodborne isolates of *S. aureus* (Jilin Entry and Exit Inspection and Quarantine Bureau) were stored at −20°C in a solution of trypticase soy broth (TSB; Qingdao Hope Biotechnology Co., Ltd) and glycerol until use. The pure cultures were transferred into TSB and cultured for 24 h under the temperature condition of 37°C for activation. Cultures were then streaked onto Baird Parker agar plates (BPA; Qingdao Hope Biotechnology Co., Ltd) and cultured for 24 h under the temperature condition of 37°C. To prepare working cultures, cells were extracted from individual colonies on the BPA plates and then placed in the TSB broth (10 ml). Bacterial cultures were cultured for 16–18 h at 37°C (Huang, 2015; Lee et al., 2015).

### 2.2 Sample preparation and bacterial inoculation

Raw pork used in the present study was purchased at a major retail supermarket in Changchun, China, and sent to the laboratory within 30 min. Pork was weighed (20 ± 0.1 g) in sterile centrifuge bottles (Hwang & Huang, 2018). Meat samples were inoculated by adding 100 μl of the *S. aureus* cocktail to the final concentration of 10^3–4^ CFU/g (Juneja et al., 2007).

### 2.3 Sample incubation and enumeration

Inoculated samples were randomly grouped and stored separately at a dynamic temperature profile and two isothermal temperature profiles (11°C, 16°C). The dynamic curves combined with two static curves were used for fitting analysis to estimate the model parameters. The dynamic temperature profile was designed by referring to a previously reported study (Huang & Hwang, 2017; Liu et al., 2019) to allow for arbitrary fluctuations and incubate samples near the minimum growth temperature (*T*_min_), thus observing the death and survival conditions of bacteria in culturing. Among the dynamic temperature profiles, 7°C could be set for observing the growth of bacteria near the minimum temperature, which is close to the estimated minimum temperature in previous reports (Lu et al., 2020; Medveďová et al., 2020). Freezing temperatures were also included in the dynamic profiles which may help accurately estimate the theoretical minimum temperature for growth (Liu et al., 2019). After the inoculation, the samples were immediately treated with 0.1% sterile peptone water (PW) by serial decimal dilutions at a preset sampling time. The diluted aliquots were spread onto the BPA base plates to estimate the bacterial populations, followed by incubation overnight at 37°C. Counts were recorded with the logarithm (log10 of CFU/g) of the subsequent dilutions. In addition, each treatment was conducted in triplicate (Kataoka et al., 2017).

### 2.4 Mathematical modeling and Estimation of kinetic parameters

For the estimation of kinetic parameters, the research then adopted a one‐step analysis method. Logistic equation of Equation (1) was used as the primary model for kinetic analysis of *S. aureus* in pork to fit survival curves. In the model, N refers to bacterial count (CFU/g), while maximum population density (*Y*_max_) (ln CFU/g) had been calculated with an upper asymptote. Equation (1) subtly portrays two cases in one equation, which were referred as previously reported (Huang & Hwang, 2017). In case the experimental temperature exceeds *T*_min_, the equation turns to the logistic equation in particular appropriate to the description of bacterial logarithmic growth having carrying capacity. Rate coefficient *K* in the case of *T* ≥ *T*_min_ is *μ*_max_ (specific growth rate), the maximum value of the first derivative on the logarithm of viable count for time (Juneja et al., 2007) (ln CFU/g per h). In case the temperature is lower than *T*_min_, the equation could be simplified as first‐order survival kinetics under *K* < 0 which can describe the inactivation behavior under unfavorable conditions (Huang & Hwang, 2017; Li et al., 2017).

(1) $$]]><fontstyle01/><!\left[CDATA\left[\begin{array}{c} \frac{\mathrm{dN}}{\mathrm{dt}}\;=\;KN\left(1‐m\times \frac{N}{e^{Y_{\max}}}\right)\\ m\;\;=\;\;0\;\text{if}\;T\;<\;T_{\min}\\ m\;\;=\;\;1\;\text{if}\;T\;\geq \;T_{\min} \end{array}\right]\right]><fontstyle01/>$$

(2) $$\begin{array}{cc} \sqrt{\mu_{\max}}=a{\left(T‐T_{\min}\right)}^{0.75} & T\geq T_{\min},K=\mu_{\max}\\ K=k(T‐T_{\min}) & T<T_{\min} \end{array}$$

In the one‐step analysis, the logistic model and the secondary model (Equation 2) were integrated for being solved concurrently through numerical analysis and modification (Huang & Hwang, 2017; Hwang & Huang, 2018). In Equation (2), $\mu_{\max}$ can be described by the Huang square root secondary model at an incubation temperature above $T_{\min}$, *a* is a regression coefficient, *T* is the incubation temperature (℃). Below $T_{\min}$, the rate (*K*) of decline in the bacterial population was also described in Equation (2), *k* as the coefficient.

Because of temperature variation during bacterial growth, the differential equation cannot be solved analytically. The fourth‐order Runge–Kutta model (Equation 3) is an effective numerical approach in solving ordinary differential equations which describe microbe dynamic growth in food (Liu et al., 2019). Kinetic parameters were estimated using nonlinear least squares optimization function, which minimizes the error or the deviation of numerical solutions from the observed data for the most suitable kinetic parameters according to laboratory growth profiles (Hwang & Huang, 2018). The numerical analysis and optimization were then made with Python (www.python.org) in combination with Numpy and Scipy.

*Y_n_* is the approximation at *t_n_* (ln CFU/g); *h* is the time step (0.1 h); f (*) is the ordinary differential equation, which is dN/dt in Equation (1); *k*_1_, *k*_2_, *k*_3_, and *k*_4_ are the increments computed by f (*) at each step.

(3) $$]]><fontstyle01/><!\left[CDATA\left[\begin{array}{c} Y_{n+1}=Y_{n}+\frac{h}{6}\left(k_{1}+2k_{2}+2k_{3}+k_{4}\right)\\ k_{1}=f\left(t_{n},\;Y_{n}\right)\\ k_{2}=f\left(t_{n}+\frac{h}{2},\;Y_{n}+\frac{h}{2}\;k_{1}\right)\\ k_{3}=f\left(t_{n}+\frac{h}{2},\;Y_{n}+\frac{h}{2}\;k_{2}\right)\\ k_{4}=f\left(t_{n}+h,\;Y_{n}+hk_{3}\right) \end{array}\right]\right]><fontstyle01/>$$

### 2.5 Model evaluation and validation

During fitting, the goodness‐of‐fit of proposed models was assessed using the root‐mean‐square error (RMSE) (Equation 4), which compares the predicted value $\hat{y}$ and the observed value y at each sampling point (Valero et al., 2018). *N* means quantity of the observed data, and P suggests quantity of parameters.

(4) $$\mathrm{RMSE}=\sqrt{\frac{\sum_{t=1}^{n}{\left(y‐\hat{y_{i}}\right)}^{2}}{N‐P}}$$

For verifying predictive models, another temperature profile set aside between 20and −20℃ was designed for the samples. Additionally, isothermal growth curves under 37, 28, 20℃, and 16℃ were also used for the forward analysis to validate the model. The accuracy factor ($A_{f}$) (Equation 5) and bias factor ($B_{f}$) (Equation 6) were applied for the validation, which performed the prediction accuracy under different conditions (Ross, 1996; Rubio et al., 2018). $A_{f}$ measures the spread between observation and model predictions, while $B_{f}$ indicates the extent of the modeled under‐ or overestimate (Rubio et al., 2018). $A_{f}$ and $B_{f}$ closer to 1 indicate the model predictions perfectly match with the experimental data. *n* represents the number of trials.

(5) $$A_{f}=10^{\frac{\sum \left|\log\left(\frac{\mathrm{predicted}}{\mathrm{observed}}\right)\right|}{n}}$$

(6) $$B_{f}=10^{\frac{\sum \log\left(\frac{\mathrm{predicted}}{\mathrm{observed}}\right)}{n}}$$

Furthermore, the residual errors (ε) were also observed to judge predictive model accuracy and figure out the law of distribution of difference between ***y*** and $\hat{y}$ (Hwang & Huang, 2018). The residual errors analysis was conducted for detecting the distribution pattern.

## 3 RESULT AND DISCUSSION

### 3.1 Survival of *S. aureus* in pork

**FIGURE 1:**
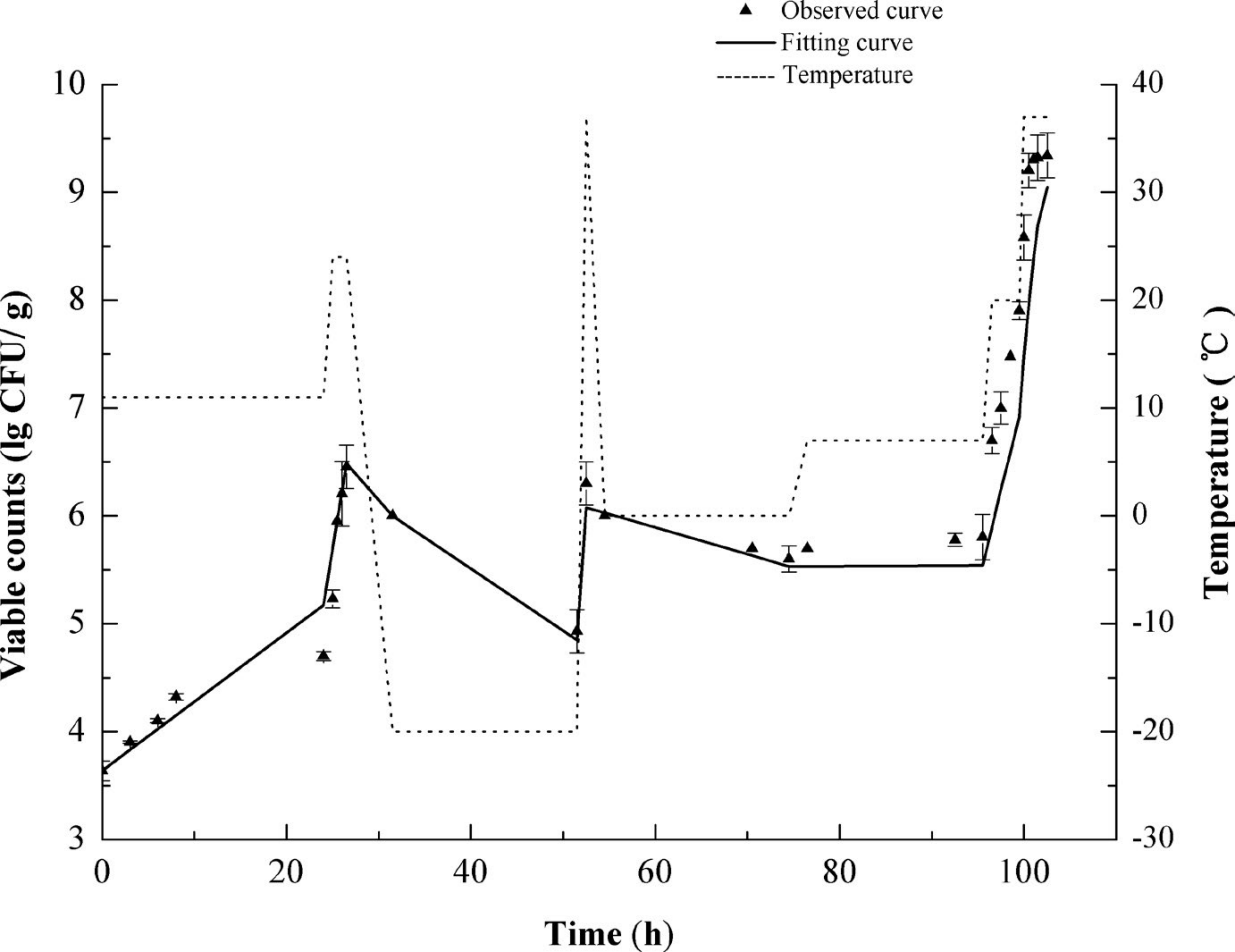
Growth and survival of *Staphylococcus aureus* in raw pork under dynamic temperature profiles and mathematical modeling. Programmed time (hours)=0, 3, 6, 8, 24, 25, 25.5, 26, 26.5, 31.5, 51.5, 52.5 54.5, 70.5, 74.5, 76.5, 92.5, 95.5, 96.5, 97.5, 98.5, 99.5, 100, 100.5, 101, 101.5, and 102.5; programmed temperature (℃) =11, 11, 11, 11, 11, 24, 24, 24, 24, −20, −20, 37, 0, 0, 0, 7, 7, 7, 20, 20, 20, 20, 37, 37, 37, 37, 37

The survival of *S*. *aureus* in pork samples was observed under dynamic nonisothermal conditions and isothermal conditions (Figure S1), and the dynamic fitting curve is shown in Figure 1. The dynamic temperature profile was set as the dotted line (Figure 1). During the incubation process, the temperature was reset to allow the random changes and observe different growth phases, simulating the temperature fluctuation that may occur during food storage and distribution. In the current study, *S*. *aureus* could survive under low temperature conditions (7℃) with atmospheric oxygen. Besides, the inactivation was observed when the samples were exposed to 0 and −20°C under the dynamic conditions.

The statistical fitting results of the model are presented in Table 1, and a good performance of the model with the observed values is illustrated. Although, growth and inactivation parameters can also be determined by a two‐step method based on isothermal experiments (Jewell, 2012). The two‐step method does not consider the gross error of parameters evaluated with primary and secondary models, which potentially provokes bias in the condition that estimates gain application in certain dynamic and extreme experimental scenarios. In contrast, the one‐step method integrates the primary model and the secondary model into one model, so as to determine gross growing and inactivating parameters in one step to fix the inverse problem efficiently (Huang, 2017). The variables of the integrated model were estimated using the data of the colony unit count versus time at different temperature profiles. The one‐step kinetic analysis is feasible to both static and nonisothermal (dynamic) conditions (Liu et al., 2019). Huang, et al. compared the results of the one‐step analysis with that of the conventional two‐step approach in *Salmonella enteritidis* growth kinetics estimate, indicated the former can produce more efficient and accurate models (Huang & Hwang, 2017). Similar results were obtained in the present study, only two constant temperature curves combined with a dynamic temperature curve were used to estimate parameters and accurate model was obtained. This approach is not only more efficient but also more accurate.

**TABLE 1:** Estimates of kinetic parameters for describing growth and survival of *Staphylococcus aureus* inoculated in pork

	Estimated value	L95CI	U95CI	Std Error	*t*‐value	*p*‐value
A	7.66 × 10^–3^	6.37 × 10^–3^	8.95 × 10^–3^	6.5 × 10^–4^	11.86	5.47 × 10^–15^
*T*_min_	6.85	5.29	8.41	0.78	8.81	3.52 × 10^–11^
*Y*_max_	21.25	20.37	22.13	0.44	48.3	1.96 × 10^–38^
*MSE*	0.24					
RMSE	0.49					
Number of observations	45					
Number of parameters	3					
Degree of freedom	42					

Note

L95CI and U95 CI, 95% confidence limit; *MSE*, mean square error; RMSE, root‐mean‐square error.

The estimated kinetic parameters of the model in this study, including *a*, *T*_min_, and *Y*_max_, are shown in Table 1. Entire estimates for parameters were at a high level of significance with *p* < .05. Numerical analysis and least squares optimization were taken to evaluate the kinetic parameter of *a*, *k*, *T*_min_, as well as *Y*_max_ from inverse analysis. The observed data extracted on dynamic and static profiles could robustly estimate parameters in the reverse analysis. The convergence for the optimization procedure was a success. All of the parameters are obtained with a very low ***p*** value except *k*, suggesting the statistical significance of *a*, *T*_min_, and *Y*_max_. The estimate of *k* (3.62 × 10^–3^) was less certain than the estimates of *a*, *T*_min_, and *Y*_max_. The estimate of *k* had a larger *p* > .05, suggesting that the regression analysis was not able to confidently estimate this parameter. One approach to solve this problem was to fix *k* to 3.62 × 10^–3^, and the data were analyzed again. This treatment did not affect the accuracy, and the same results of *a*, *T*_min_, and *Y*_max_ were obtained (Table 1). The estimated value of *a*, *T*_min_, as well as *Y*_max_ is 7.66 × 10^–3^, 6.85, and 21.25, with p values equal to 5.47 × 10^–15^, 3.52 × 10^–11^, and 1.96 × 10^–38^, respectively. Minimum growth temperature estimated by our reverse analysis approached 6.85°C, which is close to the values obtained with the Huang square root secondary model in previous reports (7℃ reported by Lu et al., 2020 and 6.06 and 7.72°C reported by Medveďová et al., 2020), which indicates that it can describe *S. aureus* growth accurately. Additionally, based on the result of kinetic analysis for *S. aureus* in raw pork, it cannot be ignored for the potential that microorganism contamination and growth at low temperatures in food. Low temperature refrigeration is seen as an effective way for food preservation. However, *S. aureus* show cold tolerance and can grow at low temperatures in raw pork, which represents a potential hazard of *S. aureus* to thrive well even after long‐term storage under low temperatures.

**FIGURE 2:**
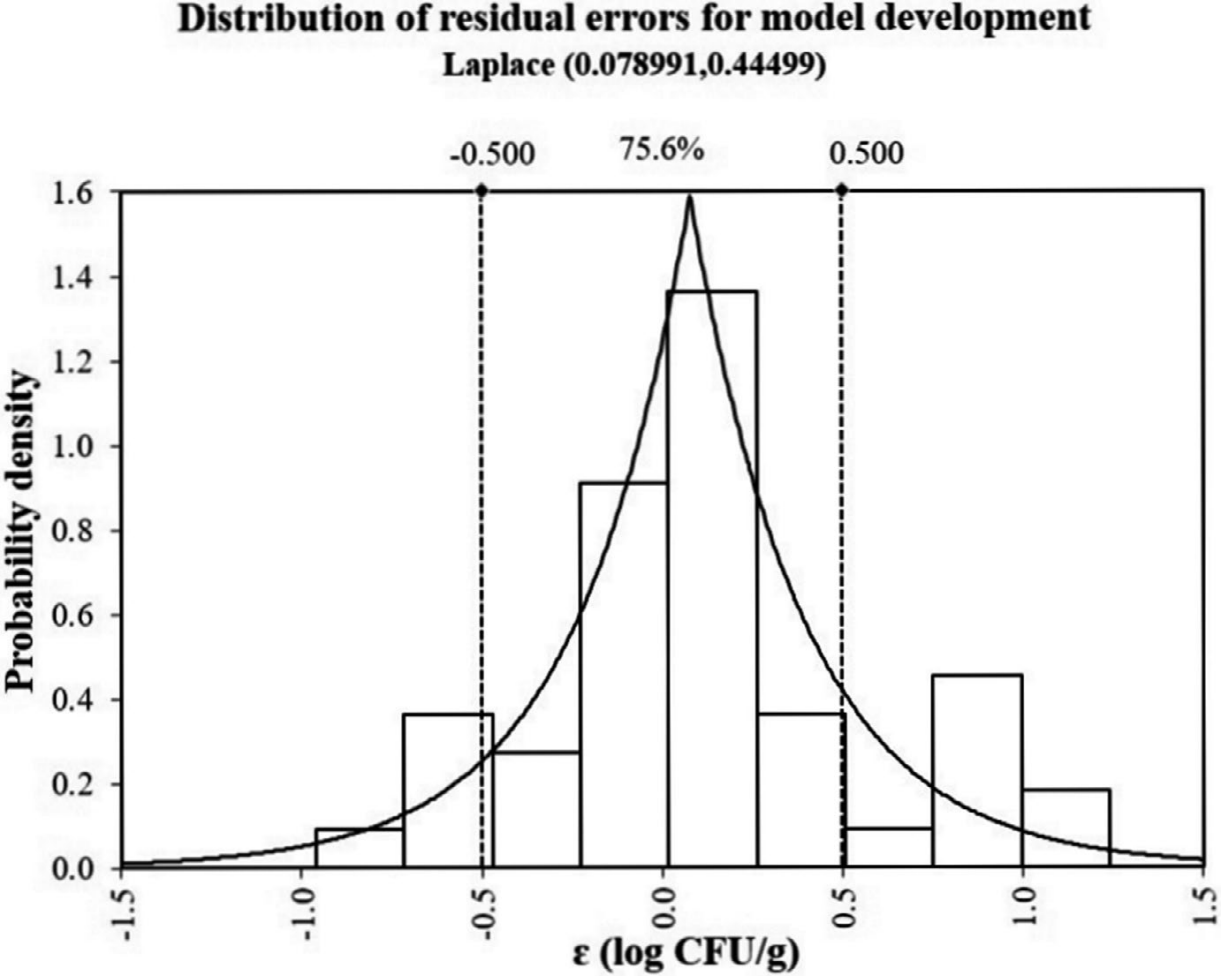
The distribution of residual errors for model development

The RMSE of the model in this study is 0.49 log CFU/g. As shown by the analysis on residual errors (ε), the residual errors abide by Laplace distribution, with the location factor as 0.08 log CFU/g (ε distribution is symmetric in ε = 0.08 log CFU/g) and the scale factor as 0.44 log CFU/g (Figure 2). This implies the exponential reduction of probability density with the growth of ε. Approximately 75.6% of prediction errors fall within ±0.5 log CFU/g, while 95.6% within ±1 log CFU/g. No estimates are less than −1 log CFU/g (under prediction), or above 1.5 log CFU/g (over prediction), suggesting that a good performance is obtained for the model fittings (Hwang & Huang, 2018).

### 3.2 Validation of predictive model

**FIGURE 3:**
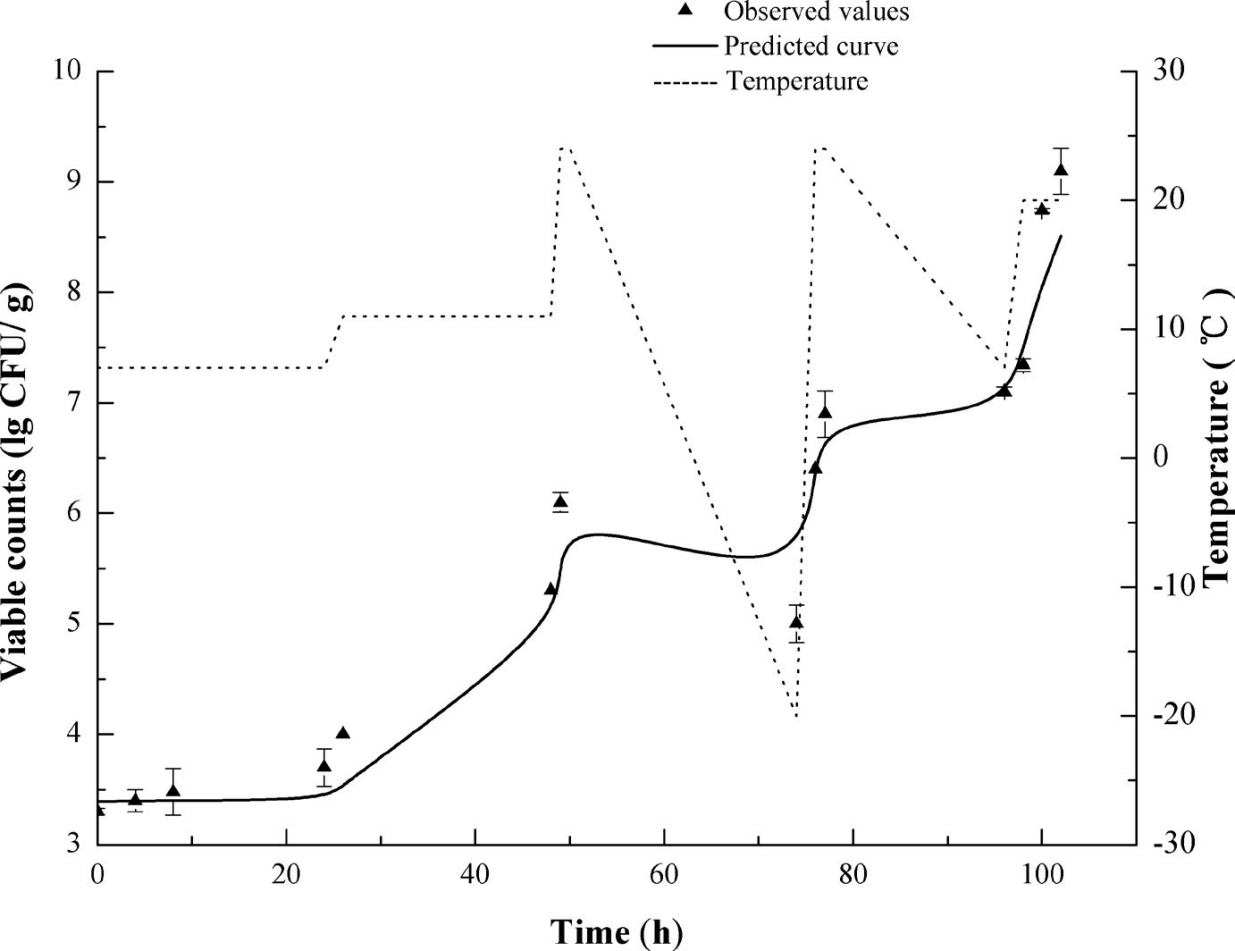
Validation of the predictive models using dynamic temperature profiles

**FIGURE 4:**
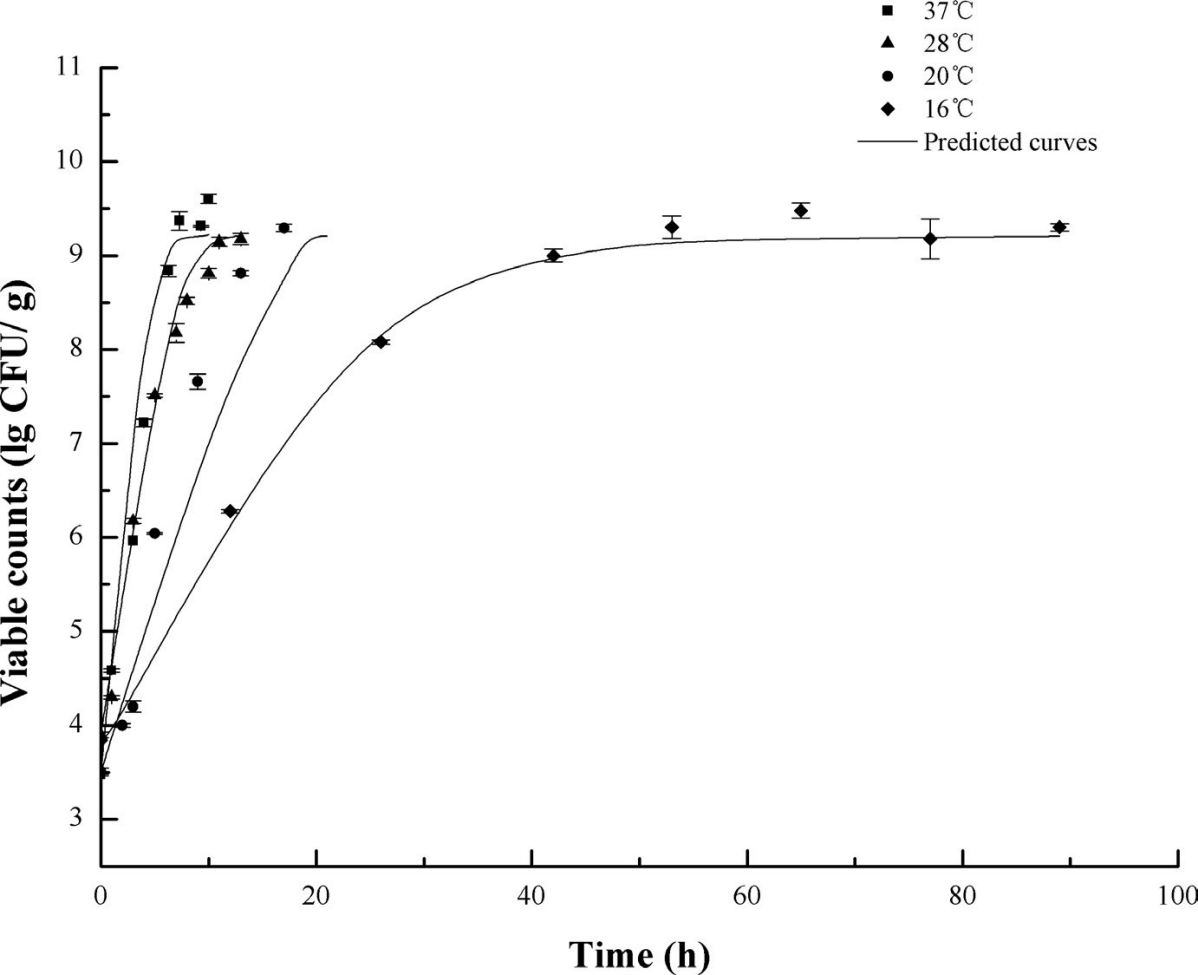
Validation of the predictive models using isothermal temperature profiles

**FIGURE 5:**
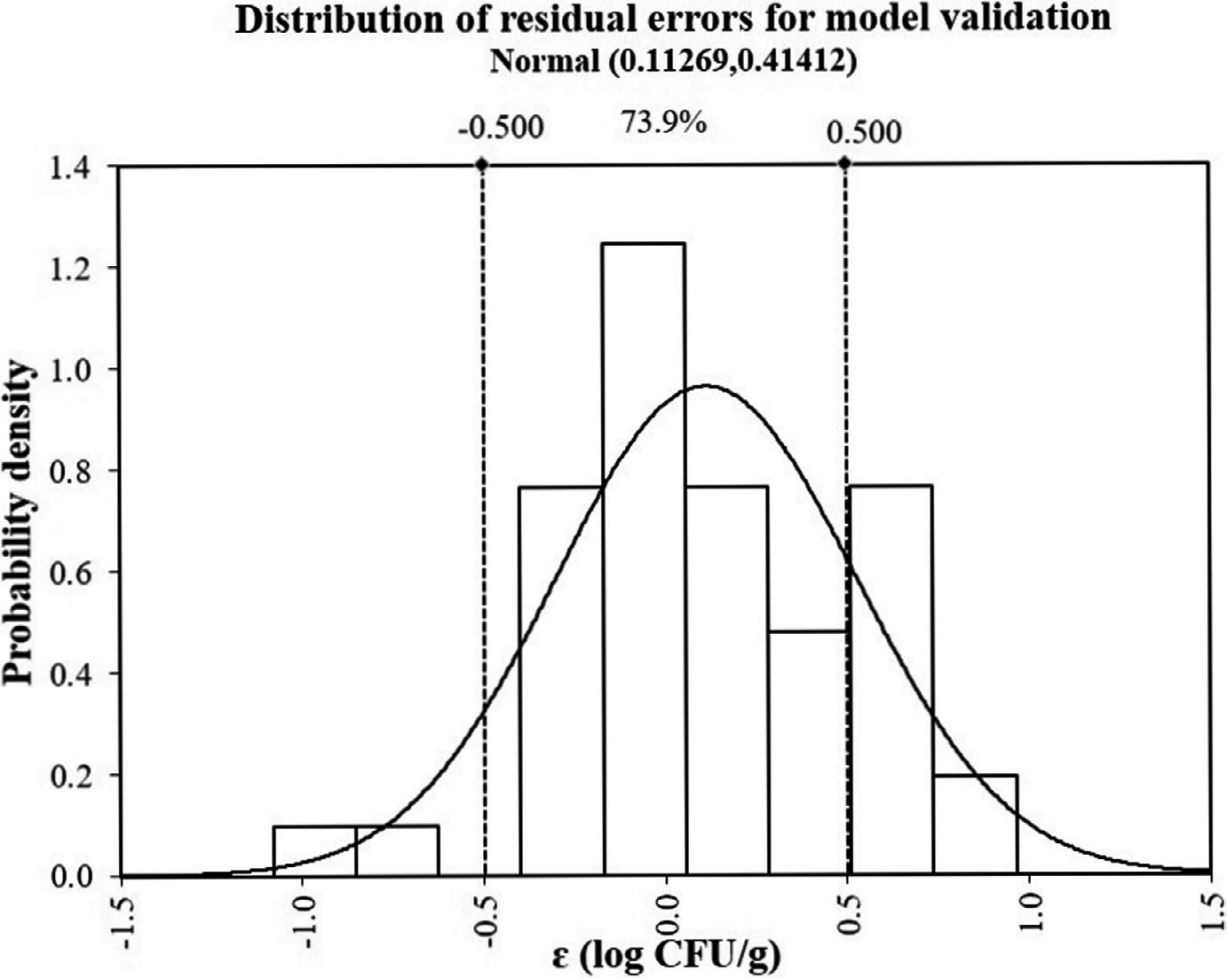
The distribution of residual errors for model validation

Another survival curve under the dynamic temperature condition and some growth curves under isothermal conditions were taken for verifying the accuracy of the proposed predictive model. The temperature profile exhibited sustained fluctuations in temperatures between −20 and 20°C, simulating arbitrary volatility of temperature conditions in frozen or refrigerated food. Since a few parts of the profile had lower than minimum growth temperature (*T*_min_), the death rate of bacteria might be in direct proportion to temperature deviation from *T*_min_. The mathematical models accurately register the growth and survival of bacteria, where model estimations were in good agreement with results of the experiment, as shown in Figure 3. Similarly, the model was also able to precisely estimate *S*. *aureus* growth under isothermal conditions at 16, 20, 28, and 37°C, which are shown in Figure 4. Predicted RMSE on dynamic and isothermal profiles is <0.44 log CFU/g, and we see from Figure 5 that the corresponding residual errors conform to the normal distribution, which is symmetric in ε = 0.1 log CFU/g. Generally, 73.9% of the prediction errors are in ±0.5 log CFU/g, and 97.8% in ±1 log CFU/g. Less than 2.2% of errors of prediction are below −1 log CFU/g (under prediction), and none are >1.0 log CFU/g. Accordingly, model prediction errors fall within the standard scope of errors, and the results of the experiment are valid. $A_{f}$ and $B_{f}$ were 1.05 and 0.98, respectively, indicating that the models were highly predictable. In an ideal case, $A_{f}$ = $B_{f}$ = 1 indicates the predictions best agree with the observed data. $A_{f}$ specifically grows from 0.1 to 0.15, so an acceptable range of $A_{f}$ is 1–1.15.

Instead of using large number of different isothermal experiments, dynamic analysis straightforwardly places microorganisms at dynamic temperatures which can induce the response by changing kinetic parameters linked to microbial growth or survival during the temperature fluctuation (Huang & Hwang, 2017; Liu et al., 2019). In our study, a dynamic temperature profile was used in combination with static profiles for fitting analysis. Interplay of varying environmental conditions and microorganisms was pondered under dynamic temperature conditions to better comprehend the physiological state of bacteria, and the result makes for the dynamic modeling of the microorganism under sophisticated ecosystems. In future researches, more accurate prediction of the foodborne pathogens in food may require the optimization of the time–temperature profiles. Meanwhile, more environmental factors could be considered for better describing the cell behavior.

In this study, an integrated model was constructed combining with the dynamic survival model and the Huang square root secondary model to describe the survival of *S*. *aureus* in raw pork under various temperature conditions. The proposed model precisely estimated the survival kinetic of *S. aureus*, which illustrates that the one‐step method has greater efficiency and accuracy in the development of kinetic models used for predicting microorganism growth and survival. Furthermore, models have been validated with good performance in the external verification and internal verification. Therefore, this model could help to set up the critical control points (CCP) on storage temperature as HACCP in meat industrial processing and distribution to improve food safety. In general, the research might provide useful information and a reliable method for the prediction of *S. aureus* contamination in pork under dynamic temperature conditions during household storage or food chain supply.

## CONFLICT OF INTEREST

The authors declare that they do not have any conflict of interest.

## ETHICAL APPROVAL

This study does not involve any human or animal testing.

## INFORMED CONSENT

Written informed consent was obtained from all study participants.

## Supporting information

Fig S1
