# Supplementary material for: Analysis and mathematical modeling of the survival kinetics of Staphylococcus aureus in raw pork under dynamic and static temperature conditions
Source: Food Sci Nutr. 2021 Oct 8;9(12):6587–95. doi: 10.1002/fsn3.2604 (PMC8645714; doi:10.1002/fsn3.2604)
Supplement: Supplementary file 1 — Fig S1 [file FSN3-9-6587-s001.docx]

A

B

Figure S1. Growth of *Staphylococcus aureus* in raw pork under static temperature conditions and mathematical modeling. (A)16℃ (B)11℃
